# Engineering chimeric human and mouse major histocompatibility complex (MHC) class I tetramers for the production of T-cell receptor (TCR) mimic antibodies

**DOI:** 10.1371/journal.pone.0176642

**Published:** 2017-04-27

**Authors:** Demin Li, Carol Bentley, Jenna Yates, Maryam Salimi, Jenny Greig, Sarah Wiblin, Tasneem Hassanali, Alison H. Banham

**Affiliations:** Nuffield Division of Clinical Laboratory Sciences, Radcliffe Department of Medicine, University of Oxford, Level 4, Academic Block, John Radcliffe Hospital, Headington, Oxford, United Kingdom; University of Nebraska-Lincoln, UNITED STATES

## Abstract

Therapeutic monoclonal antibodies targeting cell surface or secreted antigens are among the most effective classes of novel immunotherapies. However, the majority of human proteins and established cancer biomarkers are intracellular. Peptides derived from these intracellular proteins are presented on the cell surface by major histocompatibility complex class I (MHC-I) and can be targeted by a novel class of T-cell receptor mimic (TCRm) antibodies that recognise similar epitopes to T-cell receptors. Humoural immune responses to MHC-I tetramers rarely generate TCRm antibodies and many antibodies recognise the α3 domain of MHC-I and β2 microglobulin (β2m) that are not directly involved in presenting the target peptide. Here we describe the production of functional chimeric human-murine HLA-A2-H2D^d^ tetramers and modifications that increase their bacterial expression and refolding efficiency. These chimeric tetramers were successfully used to generate TCRm antibodies against two epitopes derived from wild type tumour suppressor p53 (RMPEAAPPV and GLAPPQHLIRV) that have been used in vaccination studies. Immunisation with chimeric tetramers yielded no antibodies recognising the human α3 domain and β2m and generated TCRm antibodies capable of specifically recognising the target peptide/MHC-I complex in fully human tetramers and on the cell surface of peptide pulsed T2 cells. Chimeric tetramers represent novel immunogens for TCRm antibody production and may also improve the yield of tetramers for groups using these reagents to monitor CD8 T-cell immune responses in HLA-A2 transgenic mouse models of immunotherapy.

## Introduction

Manipulating the host immune system to enable and/or to enhance anti-tumour resistance with the goal of eradicating cancer cells via immunotherapy combinations is already making an indispensible contribution to cancer treatment regimens. Cancer immunotherapy has been further revitalised by the introduction of immune checkpoint blockers (e.g. monoclonal antibodies to CTLA-4, PD-1 and PDL-1) that activate T cells, and by recent developments in chimeric antigen receptor (CAR) T-cell therapy that specifically direct effector T cells to the tumour.[1, 2]

T-cell mediated cellular immunity plays a pivotal role in tumour rejection. Through their surface T-cell receptor (TCR), T cells recognise short 9-10mer peptide epitopes presented by major histocompatibility complex (MHC) class I proteins on the surface of cells. Enhancing the numbers and activity of tumour-infiltrating lymphocytes (TILs), which are mostly composed of T lymphocytes, were among the early approaches immunologists and oncologists tested to treat cancer.[3] Monoclonal T cells and soluble T-cell receptors (TCRs) have been intensively studied for anti-tumour immunotherapy, as exemplified by Immunocore, Adaptimmune and Altor Bioscience’s soluble and membrane-associated TCR therapeutics.[4–6] The idea that targeting single epitopes derived from cancer-specific or cancer-related antigens is sufficient to treat cancer has fuelled research efforts to identify suitable epitopes and has opened up many new routes for developing novel anti-cancer immunotherapy agents.[7]

An innovative class of antibodies with binding specificities similar to that of TCRs, so-called TCR mimic, or TCR-like antibodies, has been developed in recent years to target cancer T-cell epitopes.[8, 9] These antibodies recognise cancer derived T-cell epitopes in the context of MHC-I restriction in a manner similar to that of T cells. However, instead of activating T cells to release cytokines and to mediate cell-cell contact-dependent cell killing, TCRm antibodies bind cancer cells and engage the innate immune system, including complement, natural killer cells and macrophages, to kill cancer cells. TCRm phage antibodies without the ability to engage the innate immune system have also been explored as candidates for delivery of antibody drug conjugates (ADC) because MHC-I complexes are internalised.[10]

Compared to adoptive T-cell based immunotherapy, therapeutic monoclonal antibodies have a more mature scalable manufacturing platform. They do not need to be produced for individual use, and therefore can be provided as off-the-shelf drugs for patients at much lower cost and with wider availability. Antibody therapy also allows for control of treatment dosage and the adjustment of treatment regimens depending on the patient’s responses. Multiple antibody drugs such as rituximab, bevacizumab, trastuzumab have proven utility for cancer therapy.[11]

However, the production of TCRm antibodies is a challenging process.[8, 9] A contributing factor is that the target peptide-specific epitopes only represent a small area within the whole MHC-I/peptide complex. Furthermore TCRm antibody epitopes are comprised of contributions from both the MHC-I molecule and the peptide. As with TCRs, binding to a sufficient number of amino acids within the target peptide is needed to confer antigen specificity; an example being where a TCR recognising relatively few amino acids in the target survivin peptide caused fratricide towards T cells while another with greater survivin specificity did not.[12] Either using an immunisation and hybridoma fusion approach, or phage display panning methodology, the frequencies of such TCRm antibodies are rare.[13, 14]

Widespread deregulation of the p53 tumour suppressor makes this molecule an almost universal target for cancer therapy;[15] 50% of cancers carry a *TP53* mutation while many others affect other pathway components. *TP53* mutations are highly diverse, commonly alter only a single amino acid and known examples of mutant p53-derived T-cell epitopes are scarce. Notably, vaccine strategies targeting wild type (WT) p53 have not reported damage to normal tissues[16, 17] and high-copy numbers of WT p53 peptide-MHC class I complexes were detected on tumour cells as compared to low copies on normal cells.[18–20] Therefore, to maximise applicability and avoid the necessity for mutation screening in clinical applications, we focussed our efforts on raising antibodies to peptides derived from WT p53. This also targets p53 stabilised by other mechanisms, including MDM2 overexpression, human papilloma virus infection and p14^ARF^ mutations. WT p53 is among the most highly ranked cancer antigens in the National Cancer Institute pilot project for acceleration of translational research.[21]

We successfully generated an antibody, T1-116C, that recognised the p53_65-73_ peptide (RMPEAAPPV: p53RMP) in the context of HLA-A*0201.[22] However, during the screening process the majority of the antibodies generated lacked peptide specificity and bound to MHC-I and β2m. To address this and potentially improve future TCRm production efficiency, we designed chimeric murine/human MHC-I tetramers for immunisation. Our hypothesis being that replacing the components of the MHC-1 complex, which are not directly involved in peptide presentation, with the murine ‘self’ equivalents would make these regions less immunogenic. This might then focus the immune response towards the region of the complex presenting the peptide (and/or reduce that to other regions) and thus increase the number of TCRm antibodies.

## Results

### Design of human/murine chimeric MHC-I tetramers

**Fig 1:**
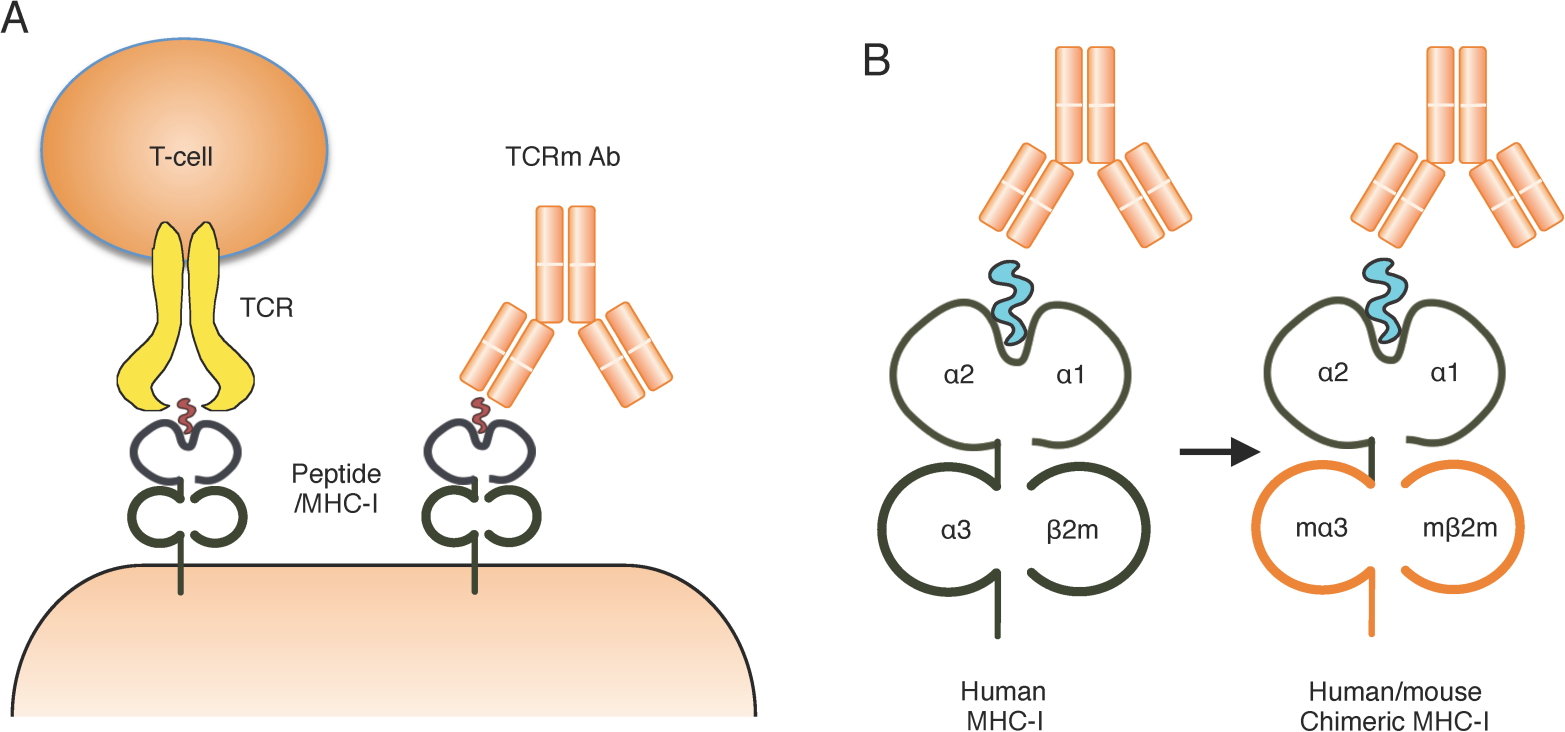
(**A**) T cell receptor mimic (TCRm) antibodies recognise short peptides, which can be derived from intracellular antigens, presented by MHC-I molecules on the cell surface. The T-cell receptor (TCR) on T cells also bind to this peptide/MHC-I complex, and this process is a critical component of normal immune surveillance. (**B**) Design of a chimeric MHC-I molecule, in which the human α3 and β2m domains are replaced with their murine counterparts (orange), while the human antigen binding α1 and α2 domains presenting the peptide remain intact.

High quality tetramers (containing an MHC-I heavy chain, an MHC-I binding peptide of typically 9–10 amino acids and β2m light chain) as an antigen for immunisation have commonly been used to successfully raise TCRm antibodies using classical hybridoma technology.[8] Both the TCR on T cells and TCRm antibodies recognise the presented peptide and the surrounding region from the α1 and α2 domains of the MHC-I molecule (**Fig 1A**). Importantly, only the α1 and α2 domains are involved in direct peptide binding, whereas the α3 domain and the β2 microglobulin (β2m) are not, but these are recognised by the humoural immune response. When using human tetramers for TCRm antibody production we found that more than half of the tetramer reactive antibodies failed to recognise the peptide and/or α1 and α2 domains (S1 and S2 Tables). Replacing the α3 and β2m domains of MHC-I with the murine equivalents has been reported to retain the tetramer’s antigen specificity for T-cells [23–26] and should therefore, in theory, provide a suitable antigen for TCRm antibody recognition.

We therefore decided to investigate whether human/mouse chimeric MHC-I tetramers in which the non-peptide-interacting components of HLA-A2 were replaced with their murine ‘self’ counterparts would improve the frequency of TCRm antibody production by exploiting immune tolerance to the murine components. Our aim was to focus the immune responses induced by the tetramers towards the peptide and α1 and α2 domains, hence increasing the probability of obtaining peptide-specific antibodies, or reducing the frequency of non-specific antibodies and thus making screening less labour intensive. The schematic in **Fig 1B** illustrates the α3 domain of human HLA-A2 being replaced with the corresponding region from murine H-2D^d^ to form a chimeric MHC-I heavy chain HLA-A2-H2D^d^, after *in vitro* refolding with murine β2m and a peptide to form a chimeric MHC-I molecule.

### Production of human/murine chimeric MHC-I tetramers

To generate MHC-I chimeric human-mouse HLA-A2-H2D^d^ tetramers, the HLA-A2-H2D^d^ chimeric heavy chain and murine β2m were separately expressed in *Escherichia coli*. The chimeric heavy chain showed poor induction of protein expression in BL21(DE3) *E*. *coli* cells (S1**A Fig**, left). Changing the IPTG concentration, induction duration and incubation temperatures did not significantly improve the yield of this chimeric heavy chain. To obtain sufficient amounts of chimeric MHC-I heavy chain protein for producing tetramers for immunisation, we optimised the codon structure of the HLA-A2-H2D^d^ coding sequence for bacterial expression (S1**B Fig**). This codon optimised cDNA sequence was subsequently cloned into the same expression vector and induced with IPTG. This codon optimisation considerably improved the production the HLA-A2-H2D^d^ chimeric heavy chain (S1**A Fig**, right).

**Fig 2:**
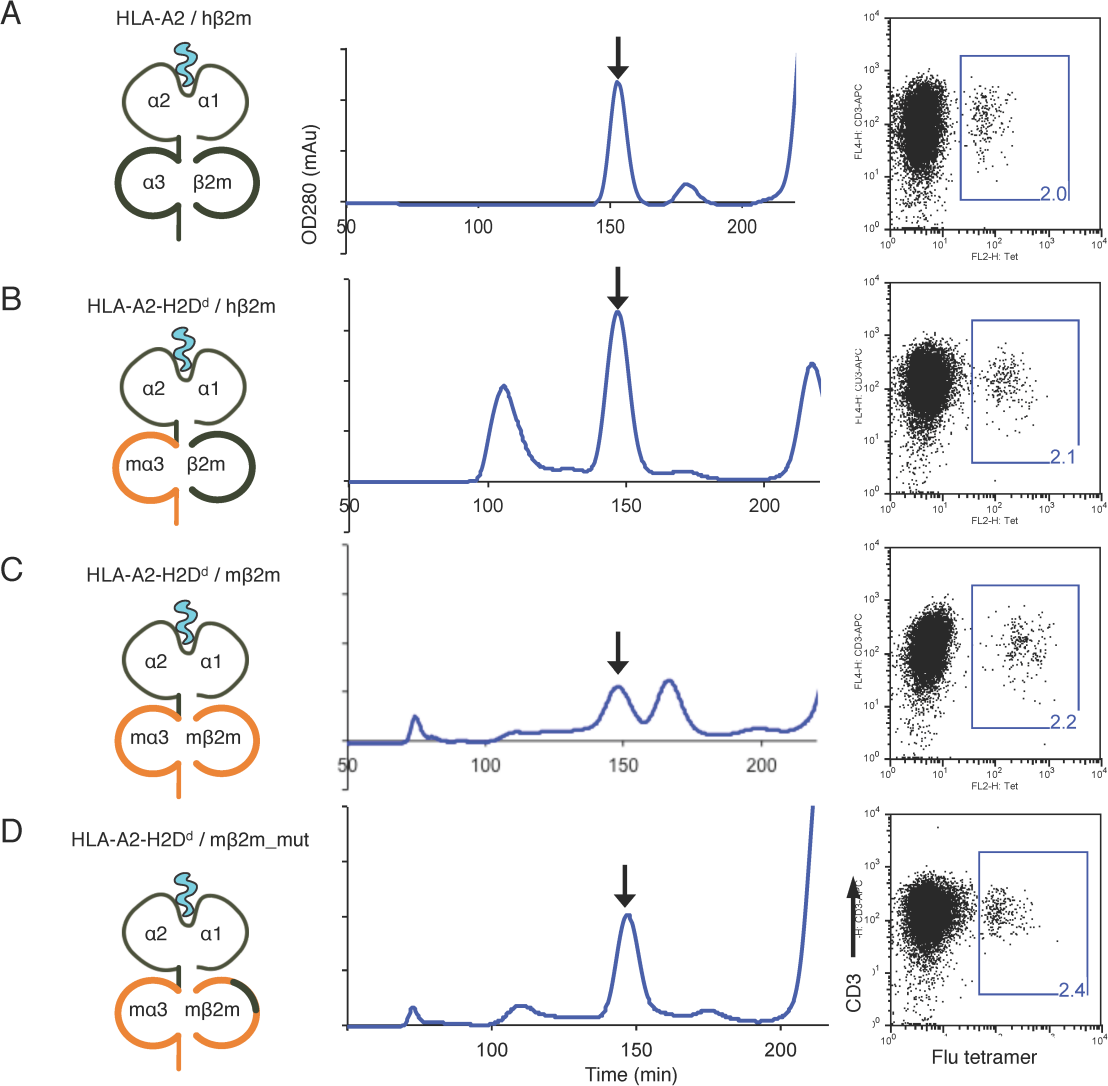
Production and validation of chimeric MHC-I tetramers. HLA-A2 (**A**) and chimeric HLA-A2-H2D^d^ heavy chains were refolded with human (**B**), mouse (**C**), or mutated mouse β2m (**D**) (hβ2m, mβ2m, and mβ2m_mut, respectively), in the presence of an influenza virus peptide GILGFVFTL (Flu). The protein refolding reactions were subsequently separated by FPLC. The arrows indicated the protein peaks containing the refolded MHC-I monomers. Two independent experiments were performed for each construct Right panels: a Flu peptide-stimulated CTL cell line was stained with human and chimeric HLA-A2 / Flu tetramers and co-stained with a CD3 antibody. This was performed three times for each tetramer and twice used a CTL line from two individuals. Gate and frequencies indicate the percentage of Flu-specific T cells bound by the respective tetramer in the T-cell line.

The ability of the HLA-A2-H2D^d^ chimeric heavy chain to be refolded with the human β2m light chain to form functional monomers was tested with a control peptide (GILGFVFTL, Flu) derived from influenza virus M1 protein (**Fig 2B**). The chimeric heavy chain showed a similar yield to that achieved with the original human HLA-A2 (**Fig 2A**). However, subsequent refolding of the chimeric heavy chain with the substitution of murine for human β2m showed only low efficiency when generating monomers with the correct conformation, as shown by FPLC (**Fig 2C**).

To improve the efficiency of forming functional chimeric MHC-I monomers containing murine β2m, we compared the crystal structures of human HLA-A2 [27] and murine H2D^d^ [28] and found that there were key differences between human and mouse β2m in their β strands 3 and 4, that are in direct contact with the α1 and α2 domains, namely the amino acids at positions 53, 54, 71 and 74. In human β2m, these positions are occupied by amino acids Ser, Asp, His and Leu, while in the mouse they are Pro, His, Met and Met, respectively. We hypothesised that because of differences between the human and mouse α1 and α2 domain structures, the changes in these β strand amino acids might disrupt the interaction between murine β2m with the human HLA-A2 α1 and α2 domains. To address this possibility we mutated these four amino acids to match those of the human β2m orthologue. When this mutated murine β2m protein was refolded with the human/murine chimeric MHC-1 heavy chain, we observed an improvement in the efficiency of monomer refolding (**Fig 2D**).

### Functional testing of chimeric MHC-I tetramers

The fully human and chimeric Flu/MHC-I monomers generated from the refolding reactions described above were tetramerised with extravidin. The tetramers were then used to stain a T-cell line that contains a subpopulation of T cells that specifically bind the Flu peptide in an HLA-A2 restricted manner. All four tetramers successfully identified the Flu-specific T-cell population with a similar frequency (**Fig 2, right panels**), indicating that the chimeric tetramers are functionally comparable to the original HLA-A2 tetramers in their ability to effectively present a peptide antigen to T cells. Therefore they are likely to be functionally equivalent to the wild type tetramers as immunogens to induce TCRm antibody responses.

### Generation of p53RMP TCRm antibodies using chimeric tetramers

**Fig 3:**
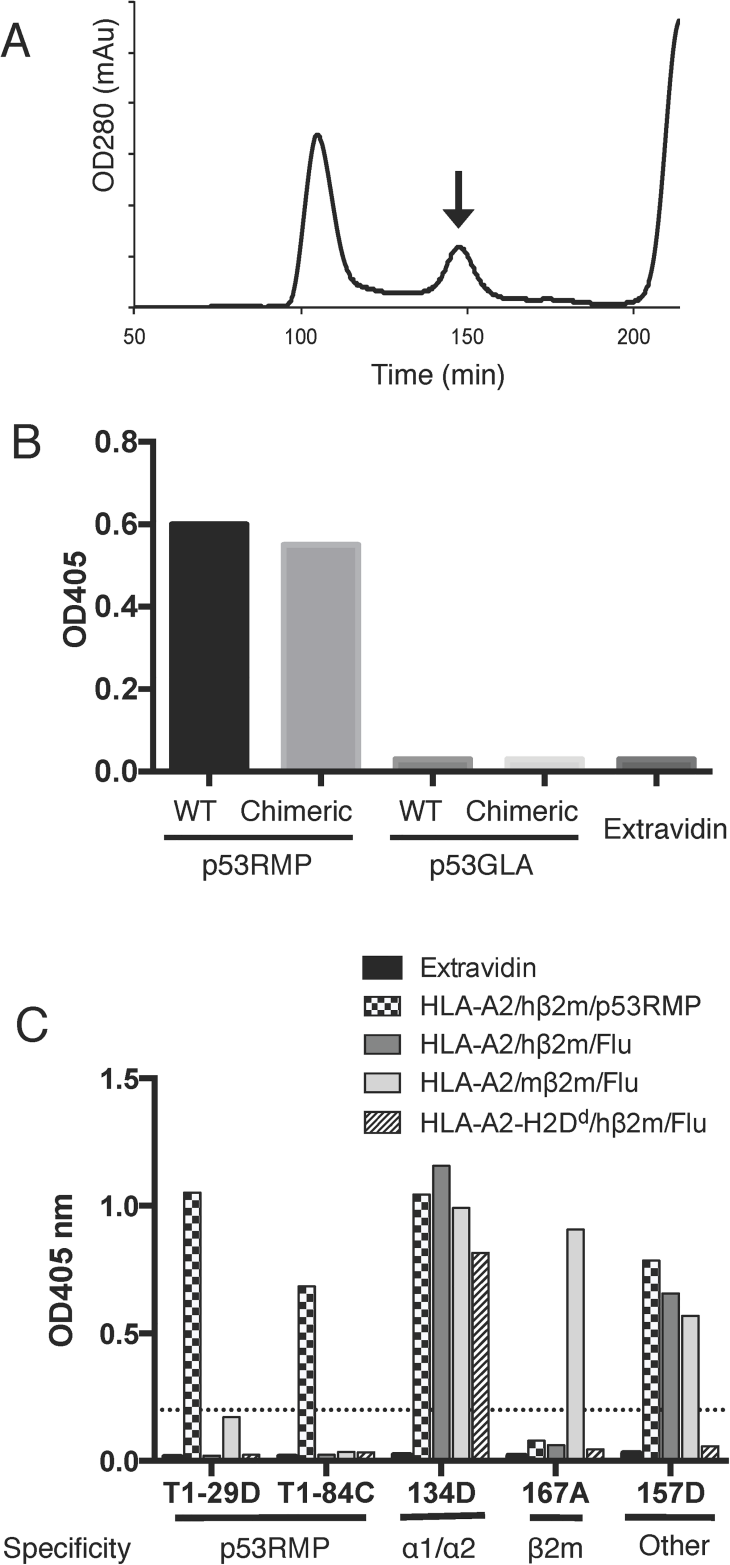
p53RMP tetramer production and fusion. (**A**) The p53RMP peptide was refolded with the HLA-A2-H2D^d^ chimeric heavy chain and mutated mouse β2m light chain, and the monomer fraction (arrow) was collected and tetramerised (n = 3) before being used as the immunogen to produce TCRm antibodies. (**B**) Validation of p53RMP monomers. Wild type (WT) or chimeric p53RMP and p53GLA monomers were immobilised onto an ELISA plate by Extravidin, and a p53RMP TCRm T1-116C was used to detect the monomers (n = 1). (**C**) Representative ELISA screening data for TCRm antibodies from chimeric tetramer fusions that were reactive with the human p53RMP tetramers used for primary screening. Hybridoma supernatants were screened against plates coated with extravidin, or extravidin tetramerised monomers comprising different combinations of HLA-A2 and HLA-A2-H2D^d^ heavy chains, human and mouse β2m, and p53RMP and Flu peptides. Hybridoma specificity was determined based on the ELISA screening results. ‘Other’ refers to an antibody where the domain specificity could not be assigned, as described in S1 Table. ELISA data illustrated is representative of three independent experiments.

Having successfully generated functional human/murine chimeric tetramers, we tested their ability to generate TCRm antibodies against tetramers containing the p53RMP-peptide and compared this with data obtained from prior immunisations with the fully human tetramer. Chimeric MHC-I monomers containing the p53RMP peptide were produced using the mutated murine β2m light chain (**Fig 3A**). A p53RMP/HLA-A2 TCRm antibody T1-116C, which was previously generated by immunising with wild type tetramers, recognised both the wild type and the chimeric complexes in an ELISA assay, suggesting the chimeric monomer was functional (**Fig 3B**). The complexes were subsequently tetramerised to form p53RMP tetramers. MF1 mice were immunised with chimeric p53RMP tetramers and after performing hybridoma fusions we identified hybridomas secreting antibodies capable of recognising the human p53RMP tetramers by ELISA. We then further investigated the domain-specificity of antibodies secreted by these initial hybridomas by testing the supernatants against tetramers with different combinations of peptides (Flu or p53RMP), and human versus murine α3 domains/β2m by ELISA. Based on the results from 2 fusions, immunisation with chimeric tetramers successfully increased the frequency of the antibodies to the human HLA-A2 α1/α2/peptide domains from <50% to nearly 88% when immunising with the chimeric tetramer, whereas those against human α3 domains/β2m were reduced from 41% to none (S2 Table). Representative ELISA screening results from later stage picked single hybridoma colonies are shown in **Fig 3C**. Two fusions with the chimeric tetramer generated two potential p53RMP TCRm antibodies, while previously three fusions with the human tetramer had generated only one.

### p53RMP TCRm antibodies showed peptide specificity in a cellular T2 assay

**Fig 4:**
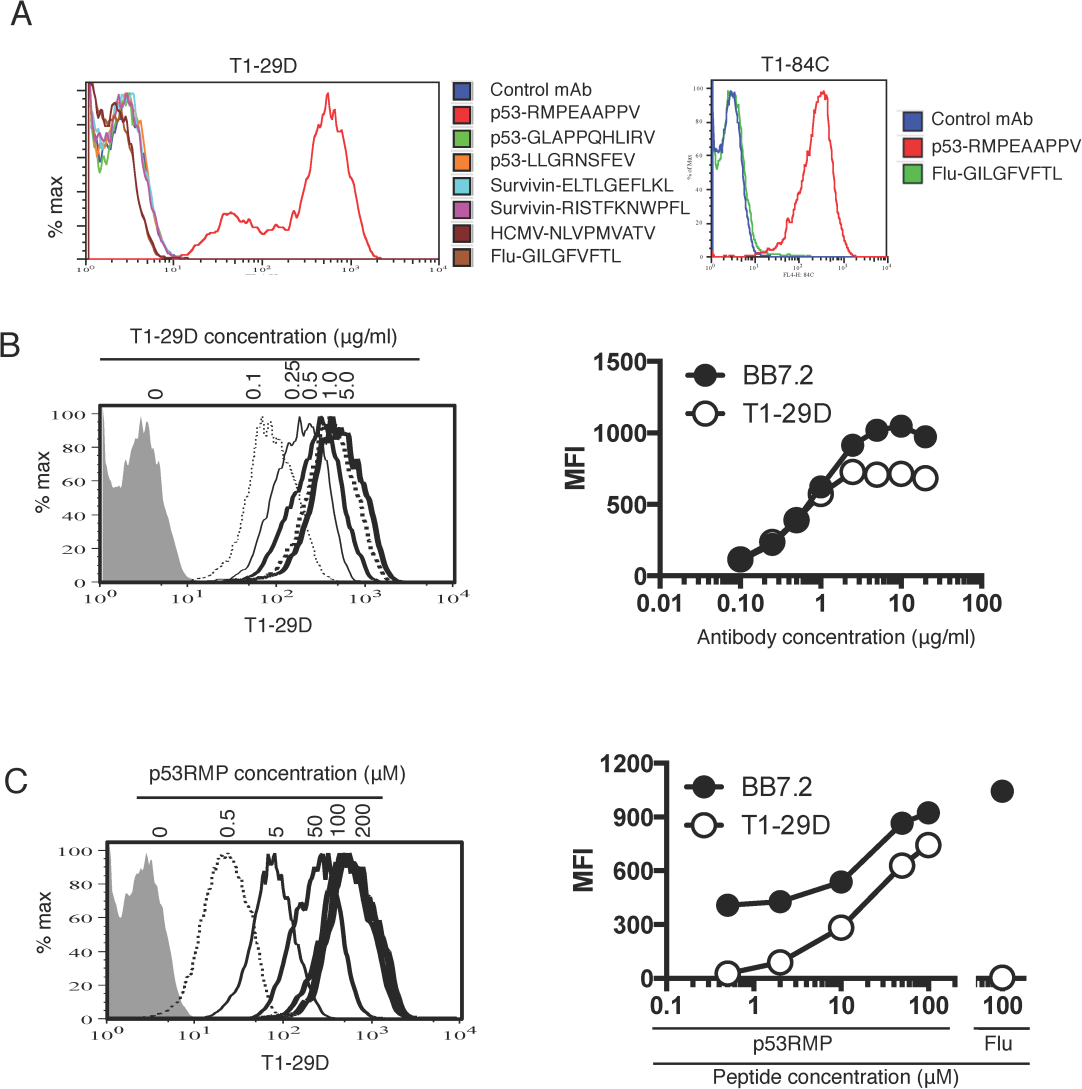
Binding of TCRm antibodies to p53RMP/HLA-A2 complexes on live cells detected by flow cytometry. (**A**) The left panel illustrates purified T1-29D mAb staining of T2 cells pulsed with p53RMP peptide as well as 2 other p53 peptides and peptides derived from survivin, HCMV and Flu (at 100μM). Staining was detected using an APC-conjugated anti-mouse secondary antibody. The T1-84C supernatant was tested only against Flu and p53RMP peptides (right panel). (**B**) T2 cells pulsed with the p53RMP peptide at 100μM were stained with the purified T1-29D mAb at the indicated concentrations. The HLA-A2 specific mAb BB7.2 was used in parallel to detect HLA-A2 expression. Mean fluorescence intensities (MFIs) of the staining were plotted on the right panel (for clarity the left panel does not contain all of the tested concentrations). (**C**) T2 cells pulsed with the p53RMP peptide at various concentrations were stained with T1-29D and BB7.2 at 10 μg/ml. MFIs of the staining were plotted on the right panel. Flu peptide pulsing at 100μM was used as a control. Data in each panel is representative of two independent experiments.

Two TCRm mAbs generated using the chimeric tetramer, T1-29D and T1-84C, were further expanded and their supernatants consistently demonstrated specificity towards the fully human p53RMP/HLA-A2 tetramer by ELISA. Culture supernatants containing the two TCRm mAbs were tested for their binding specificity to p53RMP presented by HLA-A2 on the surface of live cells. T2 cells are deficient in the transporter associated with antigen processing (TAP) and pulsing with an HLA-A2-binding peptide stabilises the HLA-A2/peptide complex on the cell surface. T1-29D and T1-84C antibodies stained the cell surface of T2 cells pulsed with the target p53RMP peptide and not T2 cells pulsed with the Flu peptide (**Fig 4A**), demonstrating their cell surface specificity for an epitope requiring p53RMP.

The T1-29D hybridoma was successfully cloned by limiting dilution and the secreted antibody was purified from the culture supernatant, as T1-84C could not be cloned or purified (despite repeated attempts) this reagent was not characterised further. Purified T1-29D was further tested for specificity of staining peptide pulsed T2 cells and showed specificity for the p53RMP peptide and did not bind other HLA-A2 presented peptides from p53 or those from unrelated antigens including survivin and HCMV (**Fig 4A**). T1-29D was tested for a dose response in its binding to the p53RMP/HLA-A2 complex on T2 cells. T2 cells pulsed with p53RMP peptide showed increased T1-29D binding when the antibody concentration was increased, and the binding was saturated at 5μg/ml (**Fig 4B**). Likewise, increasing p53RMP peptide concentrations in the T2 cell assay also increased T1-29D binding (**Fig 4C**). On both occasions, T1-29D binding was proportionally lower than that of the BB7.2 antibody, which detects the HLA-A2 expression on the cell surface independently of the peptide being presented.

### Chimeric tetramers were also used to raise p53GLA TCRm mAbs

**Fig 5:**
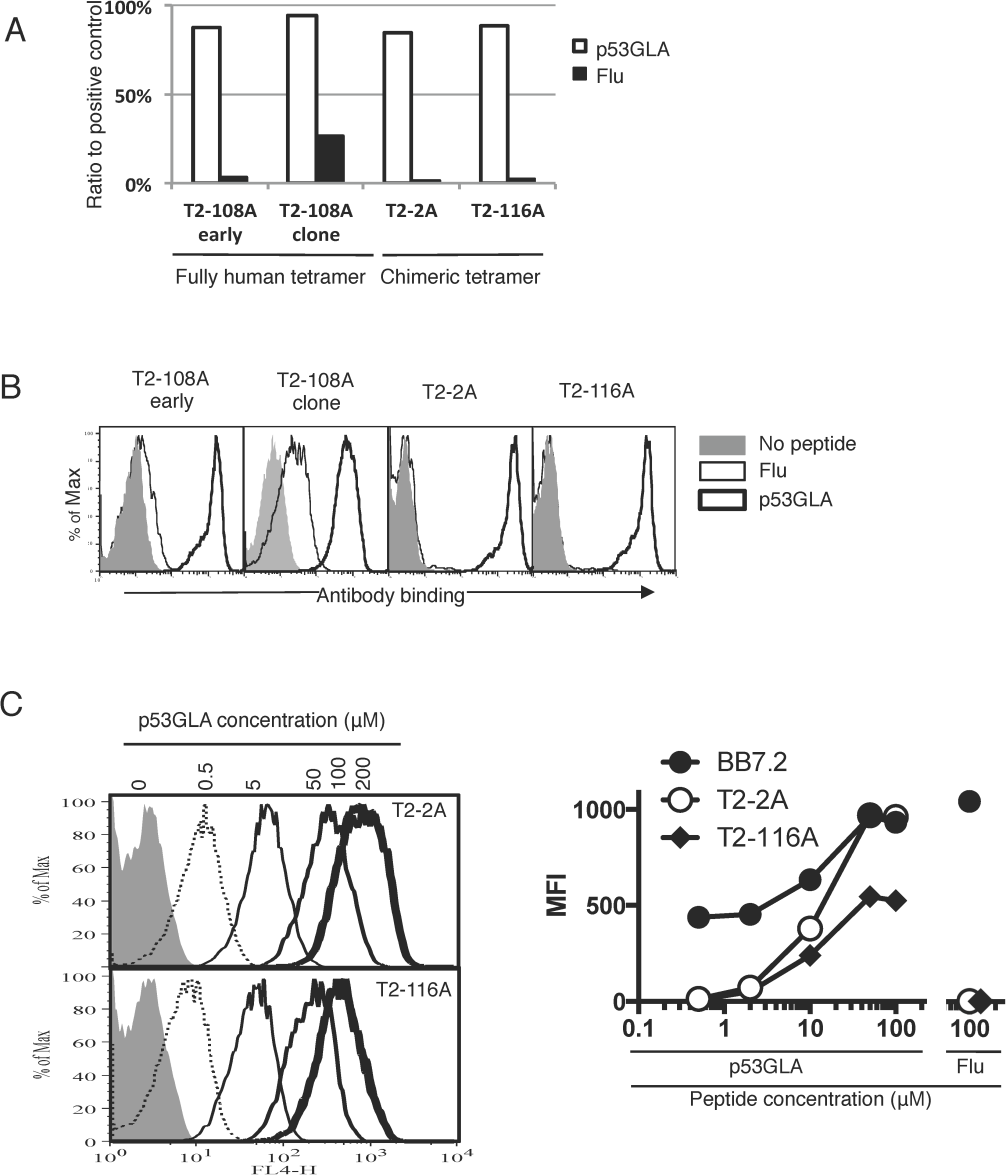
TCRm specific for p53 peptide GLAPPQHLIRV (p53GLA). HLA-A2 and chimeric HLA-A2-H2D^d^ tetramers containing the human p53GLA peptide were generated and used for TCRm antibody production. Three TCRm mAbs, of which T2-108A was generated against HLA-A2 WT tetramer and T2-2A and T2-116C against chimeric tetramers, were generated. (A) Supernatants from early culture (early) and post-cloning (clone) stage hybridoma supernatants of T2-108A as well as early T2-2A and T2-116A hybridoma supernatants were tested for their binding to p53GLA-HLA-A2 tetramers by ELISA. The Flu tetramer was used as a control. Data are representative of two independent experiments. (B) The TCRm hybridoma supernatants described above were tested for their binding to peptide-pulsed T2 cells and data were analysed by FACS. Data are representative of five independent experiments. (C) T2 cells pulsed with p53GLA peptide at various concentrations were stained with purified T2-2A or T2-116A and BB7.2 mAbs at 10 μg/ml. MFIs of the staining are plotted on the right panel. Flu peptide pulsing at 100μM was used as a control. Data are representative of two independent experiments.

Having established that chimeric tetramers could be used to produce TCRm antibodies against the p53RMP peptide, we evaluated whether this also applied to other peptides. The p53_187-197_ peptide (GLAPPQHLIRV: p53GLA) shares 100% sequence identity in humans and mice and has also been validated as a target for T-cell immunotherapy.[29] Both human and chimeric tetramers containing the p53GLA peptide were generated for TCRm antibody production. After 10 fusions (six with human and four with the chimeric tetramers), antibody T2-108A was generated from the wild type HLA-A2 p53GLA tetramer, and antibodies T2-2A and T2-116A, were raised against a chimeric p53GLA tetramer. All three hybridoma supernatants specifically recognised the p53GLA tetramers by ELISA and in the T2 presentation assay on the cell surface (**Fig 5A and 5B**). However, after hybridoma cloning, later batches of the T2-108A antibody exhibited some cross-reactivity with tetramers containing a non-target peptide (**Fig 5A and 5B**). As this was not eliminated on re-cloning the hybridoma, this reagent was not characterised any further. Both the T2-2A and T1-116A antibodies, generated using the chimeric tetramers, showed a dose response with increased binding to T2 cells pulsed with higher concentrations of the target peptide (**Fig 5C).**

## Discussion

The intracellular proteome provides a vast array of potential targets with established clinical and therapeutic relevance. The presentation of peptides derived from intracellular proteins on the cell surface by MHC-I provides an opportunity to use reagents such as TCRs and TCRm antibodies to specifically bind these epitopes. These reagents are being used to functionally understand the biology of MHC-I epitope presentation and trafficking,[30, 31] to monitor epitope presentation (for example in response to targeted immunotherapy) and as novel targeting agents/therapeutics.[8, 9]

Our aim in the current study was to investigate whether chimeric human/murine tetramers could be used to generate TCRm antibodies against two different p53 peptides and to determine whether their use increased the yield of TCRm antibodies using classical hybridoma technology. We successfully produced an HLA-A2-H2D^d^ chimeric MHC-I heavy chain and demonstrated that optimising codon usage for bacterial expression considerably increased the yield of recombinant protein. This chimeric MHC-I heavy chain could be refolded correctly with human and mouse β2m. However, introducing mutations into amino acids in murine β2m that contacted the human α1 and α2 domains to those in the human orthologue considerably improved the efficiency in MHC-I refolding, hence increasing the yield of MHC-I monomers for tetramer production. Codon optimisation and β2m mutation were needed for process scale up to generate the quantity of tetramers required for immunisation and antibody screening. These may also be useful strategies to improve the yields of chimeric tetramers for other groups who are using such reagents to monitor HLA-A2 immune responses in HLA-A2 transgenic mice, where the human tetramers fail to bind the murine T cells effectively.[23]

After immunisations and multiple hybridoma fusions, we demonstrated that using chimeric tetramers effectively reduced the numbers of hybridomas secreting antibodies against the human α3 domain of MHC-I and β2m. However, it is possible that there may also have been additional immune responses specific to the murine α3 domain of MHC-I and β2m, which would not have been detectable using a fully human tetramer for the primary ELISA screen. The number of TCRm antibodies raised against two distinct p53 target peptides remained small and thus we are unable to determine at this point in time whether the use of chimeric tetramers will significantly improve the yield of TCRm antibodies. However, of the four TCRm antibodies specifically recognising p53 that we have further characterised, three have been generated from a smaller number of fusions performed after immunisations using chimeric tetramers.

Thus chimeric tetramers represent novel immunogens for TCRm antibody production and our modifications may also improve the yield of tetramers for groups using these reagents to monitor CD8 T-cell immune responses in HLA-A2 transgenic mouse models of immunotherapy.

## Material and methods

### Animals and immunisation

The animal experiments described in this research was approved by Oxford University Animal Welfare Ethic Review Board (AWERB) and governed by appropriate UK Home Office Establishment, Project and Personal licenses. During the course of the study, the animals were checked weekly for health and welfare monitoring in additional to routine husbandry care. MF1 mice (6–8 week old females) were immunised with the tetramers following a standard protocol, i.e., each mouse was given three immunisations, with 100 μg tetramer per immunisation, at 10-day intervals. Forty days post the first immunisation, 100 μg tetramer was given as a boost immunisation, and the animals were sacrificed two days later by carbon dioxide euthanasia followed by neck dislocation. Spleen cells were harvested subsequently and fusions were performed accordingly.

### Protein expression and MHC-I monomer production

Protein expression and tetramer production are well described in the literature [32] and were performed with the following modifications. Briefly, bacterial expression constructs encoding the human HLA-A*0201 extracellular domain (amino acids 24–293), or the chimeric HLA-A2-H2D^d^ heavy chain comprising the human HLA-A2 α1-α2 domains and the murine H2D^d^ α3 domain (amino acids 24–208 of HLA-A2 fused to amino acids 185–274 of H2D^d^, cloned from BALB/c murine spleen cells), both fused with a BirA biotinylation tag (LNDIFEAQKIEWH), and separate constructs encoding human and mouse β2m (amino acids 21–119), were each generated and transformed into competent *E*. *coli* strain BL21(DE3). Protein expression was induced by addition of 0.5mM IPTG in low-salt LB medium, and insoluble inclusion bodies containing the recombinant proteins were purified using BugBuster (Merck TB245), according to the manufacturer’s instructions.

Peptides were synthesised by the peptide synthesis facility in the Weatherall Institute of Molecular Medicine (University of Oxford), these included p53 peptides p53RMP (RMPEAAPPV), p53GLA (GLAPPQHLIRV), survivin peptides ELTLGEFLKL and RISTFKNWPFL, influenza A virus M1 protein peptide (GILGFVFTL) and HCMV peptide (NLVPMVATV).

For protein refolding, an MHC-I heavy chain (15mg), β2m (12.5mg) and peptide (5mg) were added into 500ml of refolding buffer (100mM Tris.Cl pH8.0, 400mM L-Arginine, 2mM EDTA, 5mM reduced-glutathione, 0.5mM oxidised-glutathione, and 0.1mM PMSF) and refolded for 48h. The refolding complex was concentrated and buffer exchanged to 10mM Tris.Cl pH8.0, before being biotinylated with BirA protein biotin ligase (Avidity LLC BirA500) according to the manufacturer’s instructions. Biotinylated protein was then separated using an AKTA Purifier FPLC (GE Healthcare) with a Sephadex 75 column (GE Healthcare) and MHC-I/β2m/peptide monomers were isolated. Biotinylated monomers in FPLC buffer (20mM Tris.Cl pH8.0, 150mM NaCl) were aliquoted and stored at -80°C, and aliquots were thawed and tetramerised with Extravidin-PE (Sigma E4011) or Streptavidin-APC (eBioscience 17-4317-82) on use.

### Codon optimisation

The chimeric HLA-A2-H2D^d^ heavy chain fusion cDNA sequence described above was codon optimised for *E*. *coli* expression with GeneOptimizer® (ThermoFisher Scientific). The cDNA (S1**B Fig**) was subsequently synthesised by GeneArt^TM^ Gene Synthesis (ThermoFisher Scientific) and cloned into the pET9c expression vector for protein expression.

### Site-directed mutagenesis of murine β2m

The cDNA sequence encoding mature murine β2m protein (amino acids 21–119) was cloned from BALB/c murine spleen cells and introduced into the pET9c expression vector. Site-directed mutagenesis was performed using a QuikChange II Site-Directed Mutagenesis kit (Agilent #200522). The following mutations were introduced into murine β2m to improve its refolding efficiency with the chimeric MHC-I heavy chain: Pro53Ser, His54Asp, Met71His, Met74Leu. Protein expression, refolding and tetramerisation were performed similarly to human tetramers.

### Hybridoma production and screening

A standard fusion protocol was followed[33] with NS0 murine myeloma cells as the fusion partner and hybridomas were grown out under hypoxanthine, aminopterin and thymidine (HAT) selection.

Hybridoma supernatants were screened for the presence of secreted antibodies specifically, or preferentially, recognising the tetramer containing a p53 peptide rather than a control tetramer containing a peptide from influenza virus, by ELISA. Wild type HLA-A*0201 tetramers with the immunising p53 peptide or influenza peptide were screened simultaneously and individual hybridoma colonies were picked from wells where the supernatant showed enhanced binding to a tetramer containing the immunising peptide rather than control peptide. Only individual colonies secreting antibodies that specifically recognised the tetramer containing the immunising peptide were studied further and were cloned by limiting dilution. All supernatants were screened for reactivity with a wild type human tetramer, even when the immunising tetramer was chimeric.

### ELISA assay

Ninety-six well MaxiSorp plates (Sigma-Aldrich 44-2404-21) were coated with 100μl of streptavidin at 10μg/ml in PBS at 4°C overnight. The plates were then washed with PBS/0.1% Tween-20 and blocked with 1% BSA in PBS for 2h at room temperature. Plates were used fresh or kept at -20°C after washing for future use.

To test hybridoma binding to MHC-I/peptide complexes, plates were used fresh or recovered from the -20°C freezer and thawed at room temperature. MHC-I/peptide monomers were added to the wells at 1μg/ml (100μl) and incubated for 1h at room temperature. After washing with PBS-Tween 20, 100μl of mAb at 10μg/ml or neat hybridoma supernatants were added to the wells and incubated for 1h. After washing with PBS-Tween 20, HRP conjugated anti-mouse secondary antibody (Sigma-Aldrich A9044) was added at 1:1000 dilution to each well and incubated for 1h. Substrate ABTS Solution (Roche 10102946001) was added to each well (100μl) after washing and OD_405nm_ was measured with a plate reader within 5-30min.

### T2 cell binding assay

TAP-deficient T2 cells were cultured in RMPI-1640 medium with 10% foetal bovine serum supplemented with 2mM L-glutamine and 100U/ml penicillin/streptomycin at 37°C with 5% CO_2_. Cells at logarithmic phase were pulsed with peptides at 100mM (or a range of lower concentrations for peptide titration experiments) for 12h in a U-shaped bottom 96 well tissue culture plate. Cell were then harvested and stained with TCRm antibodies and/or HLA-A2-specific mAb BB7.2 (Abcam ab27728) at 10μg/ml, followed by APC conjugated goat anti-mouse secondary antibody (eBioscience 17–4010) at 1:200. Samples were washed with FACS wash buffer (2% FBS in PBS + 0.1% sodium azide) then fixed with 1% paraformaldehyde (in PBS) and acquired with a FACSCalibur (BD Bioscience).

### FACS analysis

Cells were stained with either purified TCRm antibodies, generally at 10μg/ml diluted in FACS wash buffer (2% FBS in PBS + 0.1% sodium azide), or neat hybridoma supernatants, followed by the indicated allophycocyanin (APC)-conjugated secondary antibody at 1:200 dilution: for murine primary antibodies the secondary antibody was goat anti-mouse-APC (eBioscience 17–4010); for humanised and chimeric antibodies the secondary antibody was goat anti-human IgG (H+L) secondary antibody (Jackson ImmunoResearch Laboratories 109-136-088). After washing, samples were fixed with 1% paraformaldehyde (in PBS) and acquired with a FACSCalibur (BD Bioscience). FACS data were analysed with FlowJo software (TreeStar Inc.).

### T-cell line generation and staining

The use of human tissue in this research was reviewed and approved by Oxfordshire Clinical Research Ethics Committee. Peripheral blood mononuclear cells (PBMCs) from HLA-A2^+^ healthy donors were obtained from National Health Service (NHS) Blood and Transplant. The cells were cultured in cultured in 24 well plates at 2x10^6^/well in the presence of 2μM Flu (GILGFTFVL) peptide, 5μg/ml PHA, 100U/ml IL-2, and 10% human AB^+^ serum in RPMI1640. IL-2 was supplemented every 3 days. T-cell specificity was tested by tetramer staining after day 14, when the cells were harvested by spinning at 1500rpm and stained with standard human PE-conjugated tetramers (10μg/ml) containing the Flu peptide and APC-conjugated CD3 mAb (DAKO C7225) at 37°C for 30min, before being washed with FACS wash buffer. Cells were then fixed with 1% paraformaldehyde (in PBS) and analysed by FACS as described above.

## Supporting information

S1 Fig Codon optimisation improved expression of the chimeric HLA-A2-H2D^d^ protein in E. coli. (A) Chimeric HLA-A2-H2D^d^ parental and codon optimised cDNAs cloned into an expression vector were transformed into E. coli BL21(DE3) and protein expression was induced by IPTG. Cell lysates were analysed by SDS-PAGE and Coomassie blue staining to detect protein expression, n = 4 biological replicates for expression from the parental construct and n = 3 for the codon optimised construct. The chimeric HLA-A2-H2D^d^ molecule, visible only in IPTG induced transformants expressing the codon optimised cDNA, is indicated by an arrow. (B) Codon optimised cDNA sequence encoding an HLA-A2-H2D^d^ chimera. Restriction sites are shaded; HLA-A2 α1/α2 sequence and murine H2-D^d^ α3 domain are underlined with double lines and dashed lines, respectively; the BirA biotinylation target sequence is boxed. Amino acid sequences are displayed in single letters underneath the corresponding codons, with * representing the stop codon.
(TIFF)

S1 Table ELISA reactivity used to assign domain specificity. (DOCX)

S2 Table Epitope mapping results from individual fusions. (DOCX)

## Abbreviations

ADC: antibody drug conjugate
β2m: β2 microglobulin
CAR T: chimeric antigen receptor T
IPTG: Isopropyl β-D-1-thiogalactopyranoside
MHC-I: major histocompatibility complex class I
PBMC: Peripheral blood mononuclear cell
TCR: T-cell receptor
TCRm: T-cell receptor mimic
WT: wild type
