# Supplementary figures and images for: Engineering chimeric human and mouse major histocompatibility complex (MHC) class I tetramers for the production of T-cell receptor (TCR) mimic antibodies
Source: PLoS One. 2017 Apr 27;12(4):e0176642. doi: 10.1371/journal.pone.0176642 (PMC5407768; doi:10.1371/journal.pone.0176642)

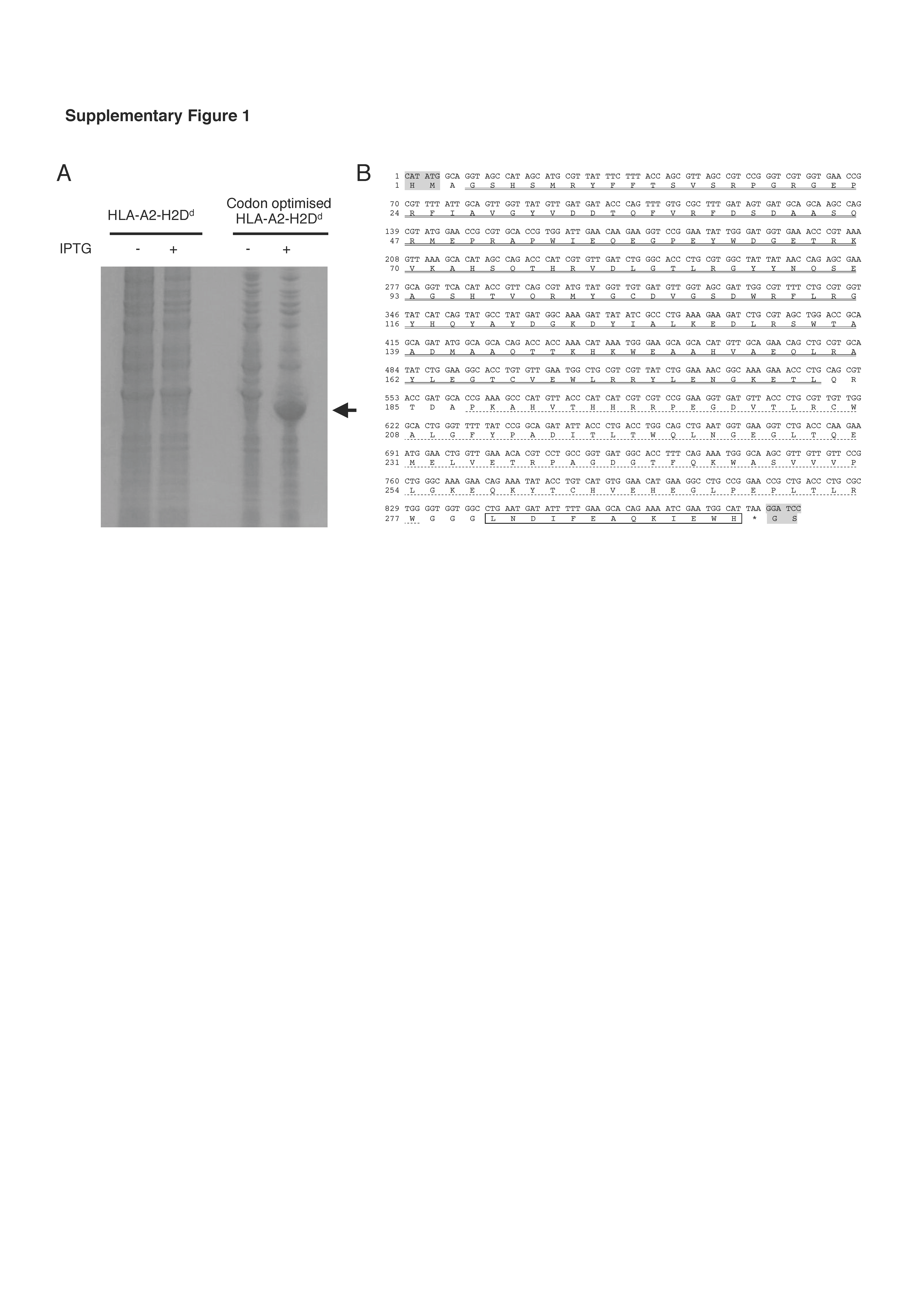

Supplement: S1 Fig — (A) Chimeric HLA-A2-H2Dd parental and codon optimised cDNAs cloned into an expression vector were transformed into E. coli BL21(DE3) and protein expression was induced by IPTG. Cell lysates were analysed by SDS-PAGE and Coomassie blue staining to detect protein expression, n = 4 biological replicates for expression from the parental construct and n = 3 for the codon optimised construct. The chimeric HLA-A2-H2Dd molecule, visible only in IPTG induced transformants expressing the codon optimised cDNA, is indicated by an arrow. (B) Codon optimised cDNA sequence encoding an HLA-A2-H2Dd chimera. Restriction sites are shaded; HLA-A2 α1/α2 sequence and murine H2-Dd α3 domain are underlined with double lines and dashed lines, respectively; the BirA biotinylation target sequence is boxed. Amino acid sequences are displayed in single letters underneath the corresponding codons, with * representing the stop codon. (TIFF) [file pone.0176642.s001.tiff]
