# Supplementary material for: Engineering chimeric human and mouse major histocompatibility complex (MHC) class I tetramers for the production of T-cell receptor (TCR) mimic antibodies
Source: PLoS One. 2017 Apr 27;12(4):e0176642. doi: 10.1371/journal.pone.0176642 (PMC5407768; doi:10.1371/journal.pone.0176642)
Supplement: S1 Table — (DOCX) [file pone.0176642.s002.docx]

**S1 Table .** ELISA reactivity used to assign domain specificity

| Plate coating |  | Extravidin | HLA-A2 hβ2m  p53 | HLA-A2 hβ2m  Flu | HLA-A2  mβ2m  Flu | HLA-A2-H2D^d^ hβ2m Flu |
| --- | --- | --- | --- | --- | --- | --- |
| Domain specificity | Peptide specific | - | + | - | - | - |
|  | Human α1α2 | - | + | + | + | + |
|  | Human α3 | - | + | + | + | - |
|  | Human β2m | - | + | + | - | + |
|  | Extravidin | + | + | + | + | + |
|  | Murine α3^1^ | - | - | - | - | + |
|  | Murine β2m^1^ | - | - | - | + | - |

The domain specificity of antibodies that did not fit these patterns was not determined and was described as 'other'.

^1^ Data is only available for the T1F5 fusion that was screened with both human and chimeric tetramers and thus enabled the identification of antibodies specific for murine β2m and murine α3.
