# Supplementary material for: Engineering chimeric human and mouse major histocompatibility complex (MHC) class I tetramers for the production of T-cell receptor (TCR) mimic antibodies
Source: PLoS One. 2017 Apr 27;12(4):e0176642. doi: 10.1371/journal.pone.0176642 (PMC5407768; doi:10.1371/journal.pone.0176642)
Supplement: S2 Table — (DOCX) [file pone.0176642.s003.docx]

**S2 Table** . Epitope mapping results from individual fusions

| Immunogen | Wild type p53 tetramer | | | | |  | Chimeric p53 tetramer | | | |
| --- | --- | --- | --- | --- | --- | --- | --- | --- | --- | --- |
| Fusion | T1F4 | T2F2 | T2F3 | Total | |  | T1F5 | T1F6 | Total | |
|  |  |  |  | No | % |  |  |  | No | % |
| Total No colonies screened^1^ | 206 | 148 | 110 | 464 |  |  | 480 | 101 | 581 |  |
| ELISA positive colonies | 21 | 15 | 14 | 50 |  |  | 434 | 7 | 441 |  |
| No Abs epitope mapped^2^ | 16 | 9 | 12 | 37 | 100% |  | 74 | 7 | 81 | 100% |
|  |  |  |  |  |  |  |  |  |  |  |
| Peptide-specific | 1 | 2 | 0 | 3 | 8% |  | 7 | 1 | 8 | 10% |
| Human α1/α2 domain | 4 | 5 | 4 | 13 | 35% |  | 61 | 2 | 63 | 78% |
| Human α3 domain | 4 | 0 | 3 | 7 | 19% |  | 0 | 0 | 0 | 0% |
| Human β2m | 4 | 1 | 3 | 8 | 22% |  | 0 | 0 | 0 | 0% |
| Extravidin | 3 | 1 | 1 | 5 | 14% |  | 0 | 4 | 4 | 5% |
| Other^3^ | 0 | 0 | 1 | 1 | 2% |  | 1 | 0 | 1 | 1% |
| Murine α3^4^ |  |  |  |  |  |  | 3 |  | 3 | 4% |
| Murine β2m^4^ |  |  |  |  |  |  | 2 |  | 2 | 2% |

^1^ ELISA against wild type p53 tetramers, with the exception of T1F5 in which both wild type and chimeric tetramers were used for initial screening.

^2^ Epitope mapping was performed retrospectively (n=1) and included those antibodies where sufficient supernatant remained after initial screening.

^3^ ‘Other’ indicates antibodies where reactivity could not be assigned to a particular domain.

^4^ Data is only available for the T1F5 fusion that was screened with both human and chimeric tetramers and thus enabled the identification of antibodies specific for murine β2m and murine α3.

No = number, Abs = antibodies, T1 refers to fusions performed with the p53RMP tetramer, T2 refers to fusions performed with the p53GLA tetramer.
